# High quality draft genome of *Lactobacillus kunkeei* EFB6, isolated from a German European foulbrood outbreak of honeybees

**DOI:** 10.1186/1944-3277-10-16

**Published:** 2015-02-19

**Authors:** Marvin Djukic, Anja Poehlein, Juliane Strauß, Fabian Jannik Tann, Andreas Leimbach, Michael Hoppert, Rolf Daniel

**Affiliations:** 1 Department of Genomic and Applied Microbiology & Göttingen Genomics Laboratory, Institute of Microbiology and Genetics, Georg-August University of Göttingen, Göttingen, Germany; 2 Department of General Microbiology, Institute of Microbiology and Genetics, Georg-August University of Göttingen, Göttingen, Germany

**Keywords:** *Lactobacillus kunkeei*, Lactic acid bacteria, European foulbrood, Honeybee, Cellular surface protein, Biofilm formation

## Abstract

The lactic acid bacterium *Lactobacillus kunkeei* has been described as an inhabitant of fructose-rich niches. Here we report on the genome sequence of *L. kunkeei* EFB6, which has been isolated from a honeybee larva infected with European foulbrood. The draft genome comprises 1,566,851 bp and 1,417 predicted protein-encoding genes.

## Introduction

Honeybees are the most economically valuable pollinators of agricultural crops [1]. A disappearance of honeybees would result in an approximately 90% decrease in production of some fruits [2]. European foulbrood (EFB) and American foulbrood (AFB) are the two most important honeybee diseases affecting the brood [3]. While the AFB is caused by the spore-forming, Gram positive bacterium *Paenibacillus larvae*[4], EFB is caused by the capsule-producing *Melissococcus plutonius*[5]. It has been shown that members of the lactic acid bacteria (LABs) inhibit the growth of *M. plutonius*[6] and *P. larvae*[7]. LABs are found in a variety of habitats, including human and animal microbiomes, and are used as food additives.

The honeybee crop microbiome consists of 13 bacterial species belonging to the genera *Lactobacillus* and *Bifidobacterium*[8]. These bacteria play a key role in the production of honey and bee bread. The latter serves as long-term food storage for adult honeybees and larvae. *L. kunkeei* is a common symbiont for *Apis* and the dominating LAB member in bees [6]. The organism is a specialist for colonization of the honeybee crop and interacts with the epithelial layer of the crop. *L. kunkeei* has been described as a fructophilic LAB [9]. Initially, it was isolated from wine [10], but it has also been found on flowers and in honey.

*L. kunkeei* EFB6 is the first LAB isolated from a German EFB-diseased larva. Here, we describe genomic features of this organism, focusing on factors that improve competition with bacteria such as *M. plutonius* and *P. larvae**.* In addition, potential cell surface proteins that might play a role in cellular adhesion and biofilm formation are analyzed.

### Organism information

In October 2012, an EFB outbreak in Bavaria (Germany) was confirmed. EFB-diseased larvae from this outbreak were collected, immediately frozen in liquid nitrogen and stored at -80°C for further investigation. Several EFB-infected larvae were dissected under sterile conditions. To obtain LAB the guts of the larvae, which formed a yellow, glue-like slime, were suspended in MRS medium (Carl Roth GmbH & Co KG, Karlsruhe, Germany) and subsequently streaked on solidified MRS to isolate single colonies. Strain *L. kunkeei* EFB6 (Table 1, Additional file 1: Table S1) was isolated from these agar plates after aerobic incubation at 35°C.

**Table 1:** **Classification and general features of *Lactobacillus kunkeei *** **EFB6**

**MIGS ID**	**Property**	**Term**	**Evidence code**
	Classification	Domain *Bacteria*	TAS [11]
		Phylum *Firmicutes*	TAS [12-15]
		Class *Bacilli*	TAS [16]
		Order *Lactobacillales*	TAS [17]
		Family *Lactobacillaceae*	TAS [18]
		Genus *Lactobacillus*	TAS [18-21]
		Species *Lactobacillus kunkeei*	TAS [10]
		strain: EFB6	TAS (this study)
	Gram stain	Positive	TAS [10]
	Cell shape	Rod-shaped	IDA
	Motility	Non-motile	IDA
	Sporulation	Non-sporulating	NAS
	Temperature range	Mesophile	TAS [10]
	Optimum temperature	30°C	NAS
	pH range; Optimum	4.5-6.2; 6	NAS
	Carbon source	Varied	NAS
MIGS-6	Habitat	Honeybee larva	IDA
MIGS-6.3	Salinity	5% NaCl (w/v)	TAS [10]
MIGS-22	Oxygen requirement	Facultative	IDA
MIGS-15	Biotic relationship	Host-associated	TAS [6]
MIGS-14	Pathogenicity	Non-pathogen	NAS
	Biosafety level	1	TAS [22]
MIGS-23	Isolation	EFB-diseased honeybee larva	IDA
MIGS-4	Geographic location	Bavaria, Germany	IDA
MIGS-5	Sample collection	October 1, 2012	IDA
MIGS-4.1	Latitude	49°14′ N	IDA
MIGS-4.2	Longitude	11°05′ E	IDA
MIGS-4.4	Altitude	400 m a.s.l	IDA

**Figure 1:**
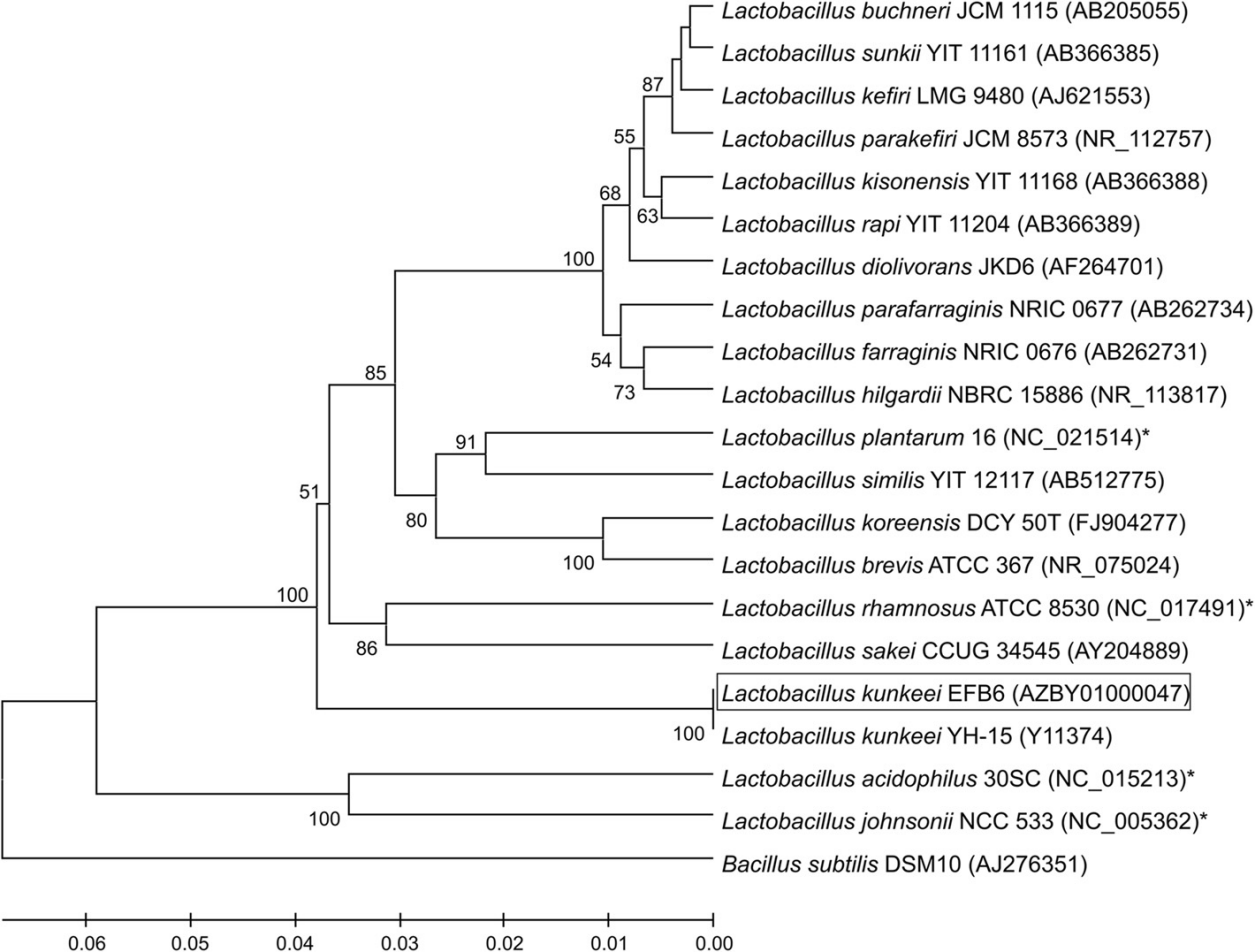
**Phylogenetic tree highlighting the position of *L. kunkeei *EFB6 relative to other *Lactobacillus *strains based on 16S rRNA gene sequences.** GenBank accession numbers are indicated in parentheses. Asterisks indicate that a consensus sequence was calculated from all 16S rRNA gene sequences present in the corresponding genome. *L. kunkeei* EFB6 is boxed. Sequences were aligned using ClustalW 1.6 [25]. The phylogenetic tree was obtained by using the UPGMA method within MEGA 6.06 software [26]. Numbers at nodes are bootstrap values calculated from 1,000 resamplings to generate a majority consensus tree. *Bacillus subtilis* DSM10 was used as outgroup. The scale bar indicates the nucleotide sequence divergence.

**Figure 2:**
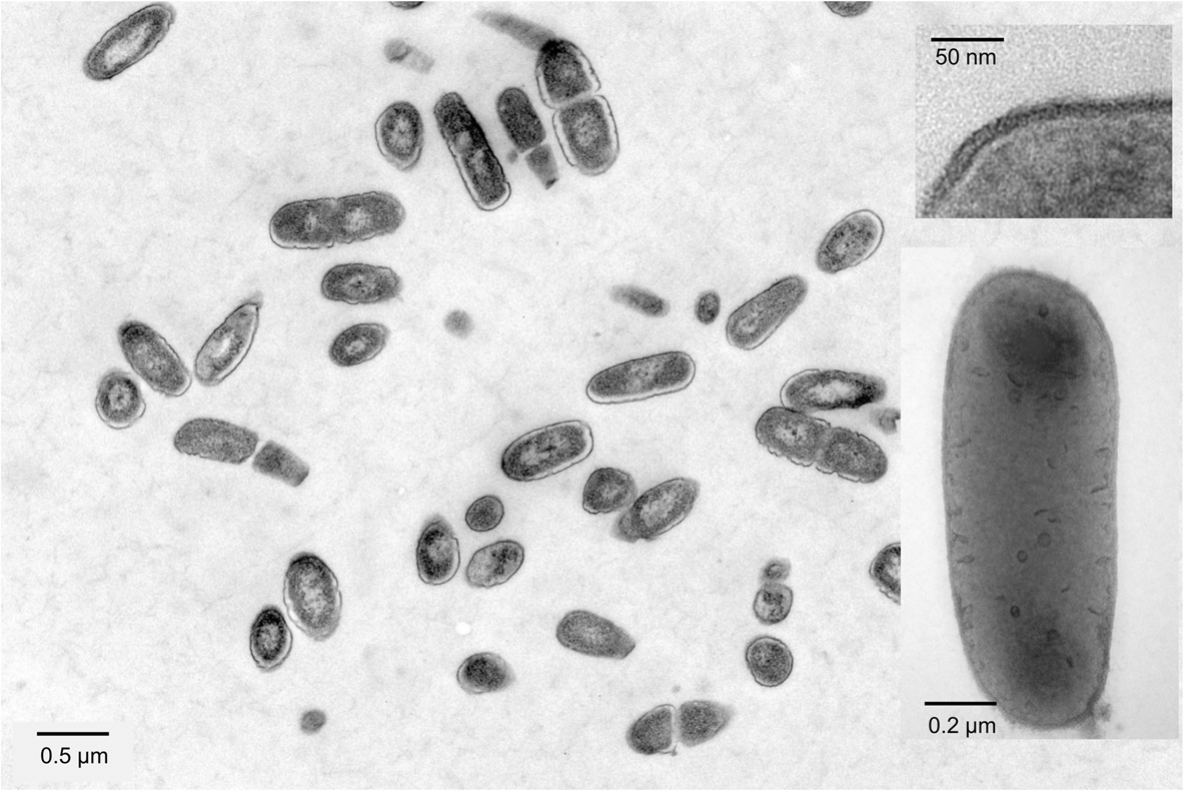
**Electron microscopy of *L. kunkeei *EFB6.** Large image and upper right inset: stained ultrathin sections; lower right inset: negatively stained single cell (staining salt: uranyl acetate, 4 %, w/v).

*L. kunkeei* EFB6 is a non-sporulating, low G + C Gram positive member of the *Lactobacteriaceae* and taxonomically related to the genus *Pediococcus*. The strain exhibited a 100% 16S rRNA gene nucleotide sequence identity to the type strain *L. kunkeei* YH-15 (Table 1, Figure 1). Cells harvested in exponential growth phase exhibited a length ranging from 0.7 to 1.3 μm and a diameter ranging from 0.3 to 0.5 μm as determined by transmission electron microscopy (TEM) of either negatively stained or ultrathin-sectioned samples (Figure 2). Preparations for ultrathin sectioning and negative staining of cells were performed as described by [23]. The *L. kunkeei* EFB6 cell wall is approximately 12 nm thick. This value is rather thin compared to cell walls of other Gram positives [24]. Three distinct wall layers of *L. kunkeei* EFB6 (two darker stained outer and inner layers and a brighter layer in between) could be distinguished by TEM. Surface layers and cellular appendages (pili, fimbriae) were not detected.

## Genome sequencing and annotation

### Genome project history

The organism was selected for sequencing on the basis of its use as potential inhibitor for the primary agents of AFB and EFB [6,7]. The aim was to investigate potential factors to increase bacterial competition fitness and cell surface proteins, which might be important for cellular adhesion and biofilm formation.

A summary of the project information is shown in Table 2.

**Table 2:** Genome sequencing project information

**MIGS ID**	**Property**	**Term**
MIGS-31	Finishing quality	Improved high-quality draft
MIGS-28	Libraries used	One Illumina paired-end library with1 kb insert size
MIGS-29	Sequencing platforms	Illumina GAII
MIGS-31.2	Fold coverage	142.96 × Illumina
MIGS-30	Assemblers	SPAdes 2.5
MIGS-32	Gene calling method	YACOP, Glimmer
	Locus Tag	LAKU
	Genbank ID	AZBY00000000
	GenBank Date of Release	May, 2014
	GOLD ID	Gi0053745
	NCBI project ID	227106
	BIOPROJECT	PRJNA227106
	Project relevance	Host-associated

### Growth conditions and DNA isolation

To isolate genomic DNA *L. kunkeei* EFB6 was grown aerobically in 50 ml MRS medium at 35°C with shaking at 150 rpm (Lab-Therm Lab-Shaker, Adolf Kühner AG, Birsfelden, Switzerland). Cells were harvested in exponential growth phase using a Beckman Coulter Allegra™ X-12R centrifuge (Beckman Coulter GmbH, Krefeld, Germany) for 25 minutes at 2,750 *g* and 4°C. Genomic DNA was isolated using the Epicentre® MasterPure™ DNA Purification kit (Epicentre®, Madison, WI, USA).

### Genome sequencing and assembly

Whole-genome sequencing of *L. kunkeei* EFB6 was performed by employing the Genome Analyzer II (Illumina, San Diego, CA). The shotgun library was prepared according to the manufacturer’s protocols. For *de novo* assembly, we used 2,000,000 paired-end Illumina reads (112 bp) and the SPAdes 2.5 software [27]. The final assembly contained 55 contigs larger than 500 bp and revealed an average coverage of 142.96.

### Genome annotation

For automatic gene prediction the software tools YACOP [28] and Glimmer [29] were used. Identification of rRNA and tRNA genes was performed by employing RNAmmer [30] and tRNAscan [31], respectively. The annotation provided by the IMG-ER system [32] was corrected manually. For this purpose, data obtained from different databases (Swiss-Prot [33], TrEMBL [34] and InterPro [35]) were used to improve the quality of the annotation.

## Genome properties

The genome statistics are provided in Table 3. The high quality draft genome sequence consists of 55 contigs that account for a total of 1,566,851 bp and a G + C content of 37 mol%. Of the 1,455 predicted genes, 1,417 were putatively protein-encoding, 35 represented putative tRNA genes and three putative rRNA genes. For the majority of the protein-encoding genes (75%) a function could be assigned. The distribution of these genes into COG functional categories [36] is shown in Table 4.

**Table 3:** Genome statistics

**Attribute**	**Value**
Genome size (bp)	1,566,851
DNA coding (bp)	1,413,077
DNA G + C (bp)	578,359
DNA scaffolds	55
Total genes	1,455
Protein coding genes	1,417
RNA genes	38
Pseudo Genes	0
Genes in internal clusters	20
Genes with function prediction	1,012
Genes assigned to COGs	1,195
Genes assigned Pfam domains	1,221
Genes with signal peptides	62
Genes with transmembrane helices	419
CRISPR repeats	0

**Table 4:** Number of genes associated with the general COG functional categories

**Code**	**Value**	**% age**	**Description**
J	137	10.57	Translation, ribosomal structure and biogenesis
A	0	0.00	RNA processing and modification
K	95	7.33	Transcription
L	94	7.25	Replication, recombination and repair
B	0	0.00	Chromatin structure and dynamics
D	24	1.85	Cell cycle control, cell division, chromosome partitioning
V	18	1.39	Defense mechanisms
T	32	2.47	Signal transduction mechanisms
M	88	6.79	Cell wall/membrane biogenesis
N	10	0.77	Cell motility
U	25	1.93	Intracellular trafficking and secretion
O	45	3.47	Posttranslational modification, protein turnover, chaperones
C	49	3.78	Energy production and conversion
G	67	5.17	Carbohydrate transport and metabolism
E	112	8.64	Amino acid transport and metabolism
F	68	5.25	Nucleotide transport and metabolism
H	34	2.62	Coenzyme transport and metabolism
I	35	2.70	Lipid transport and metabolism
P	61	4.71	Inorganic ion transport and metabolism
Q	13	1.00	Secondary metabolites biosynthesis, transport and catabolism
R	155	11.96	General function prediction only
S	134	10.34	Function unknown
-	260	17.87	Not in COGs

**Table 5:** Primer used in this study

**Primer**	**DNA sequence (5′-3′)**	**Open reading frame**	**Product size**
LKU_ORF1A_for	AACCAAGAGTAACGATGCCC	LAKU_4c00030	536 bp
LKU_ORF1A_rev	CTTTGGTAATCGGCTTGTGC		
LKU_ORF1B_for	CGATGCACAAACTGCTTACG	LAKU_4c00030	355 bp
LKU_ORF1B_rev	CATCCTTTTGTGCGTCGTTG		
LKU_ORF2_for	AGCTCTTTTAGGTGCGTCTG	LAKU_4c00040	323 bp
LKU_ORF2_rev	TATGCGTCTTGGTGGTTTGC		
LKU_ORF3_for	GCGACTTTGTCTGTTTTGGG	LAKU_4c00050	358 bp
LKU_ORF3_rev	ATAGCCCCAGCATATCCAGC		
LKU_ORF4_for	CTACGTTGAGGTTTCCGCTC	LAKU_4c00060	566 bp
LKU_ORF4_rev	GTTGGAGTTACCTTGCCACC		
LKU_ORF5_for	TCCCAGTAGTAACAAGTAACACC	LAKU_4c00070	358 bp
LKU_ORF5_rev	AAGCGGTTGATTTCCATTGAC		

### Insights into the genome

**Figure 3:**
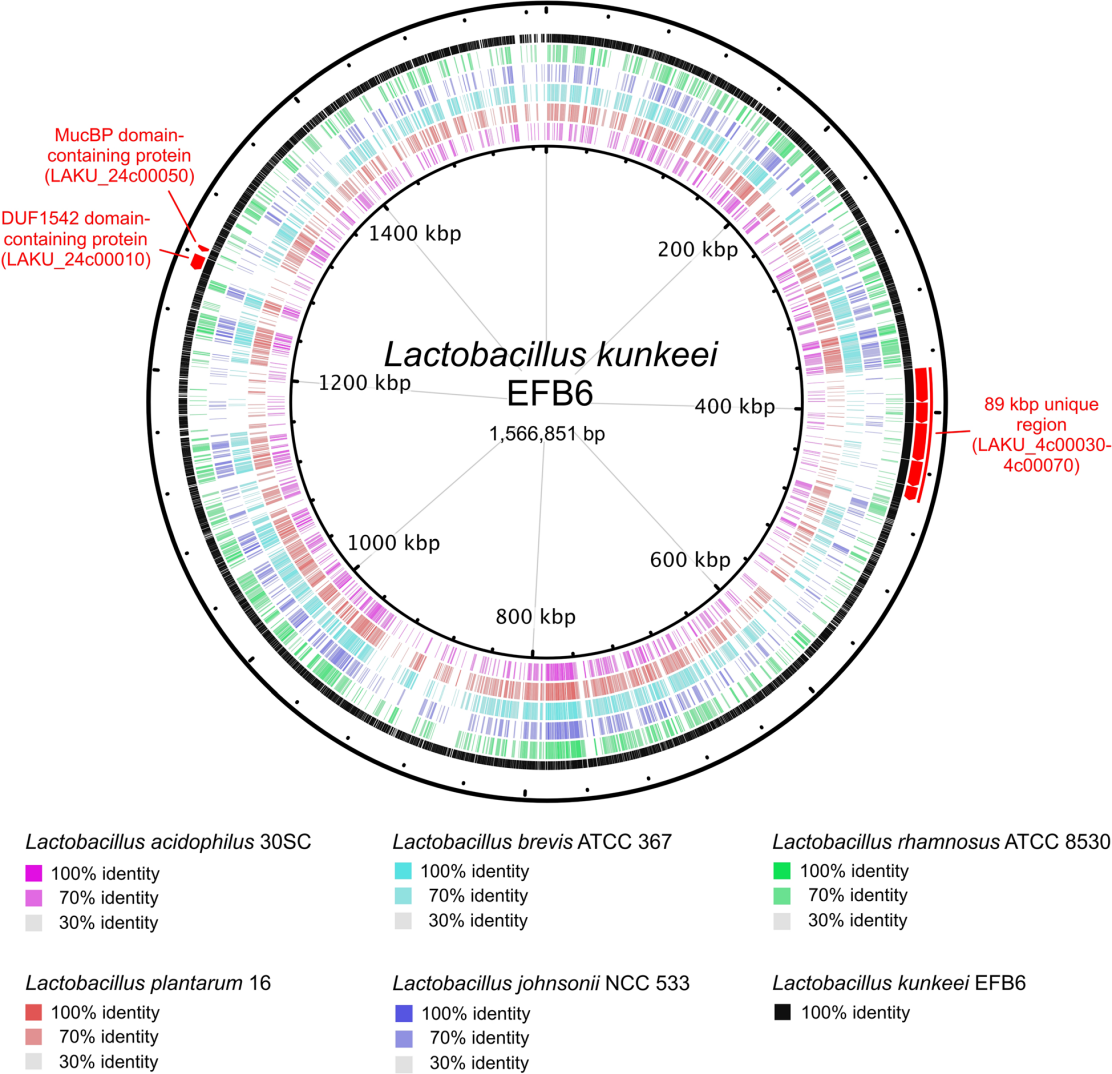
***L. kunkeei *EFB6 artificial circular chromosome map.** Comparisons (blastp) of *L. kunkeei* EFB6 chromosome to *Lactobacillus acidophilus* 30SC (NC_015213), *Lactobacillus plantarum* 16 (NC_021514), *Lactobacillus brevis* ATCC 367 (NC_008497), *Lactobacillus johnsonii* NCC 533 (NC_005362), and *Lactobacillus rhamnosus* ATCC 8530 (NC_017491), using the BRIG software [38] are shown in black, purple, red brown, cyan, blue and green, respectively. Gene regions used for detailed analyses are depicted in an outer circle and marked in red.

Five different *Lactobacillus* species were used for genome comparisons with *L. kunkeei* EFB6 based on blastp [37]. Results are shown in Figure 3. All five species are of interest as probiotics, part of the gastrointestinal tract of animals or humans, or used in the production of fermented food. 

The identification of orthologous proteins was performed with the program Proteinortho 5.04 [39] by using the protein content deduced from 232 lactobacilli genomes as references (GenBank database as of 28.02.2014). For this purpose ncbi_ftp_download v0.2, cat_seq v0.1 and cds_extractor v0.6 were used [40]. With an identity cutoff of 50%, we identified 425 proteins in *L. kunkeei* EFB6 without orthologs in any other *Lactobacillus* species. Among these unique *L. kunkeei* EFB6 proteins, we selected 7 proteins for detailed analyses.

**Figure 4:**
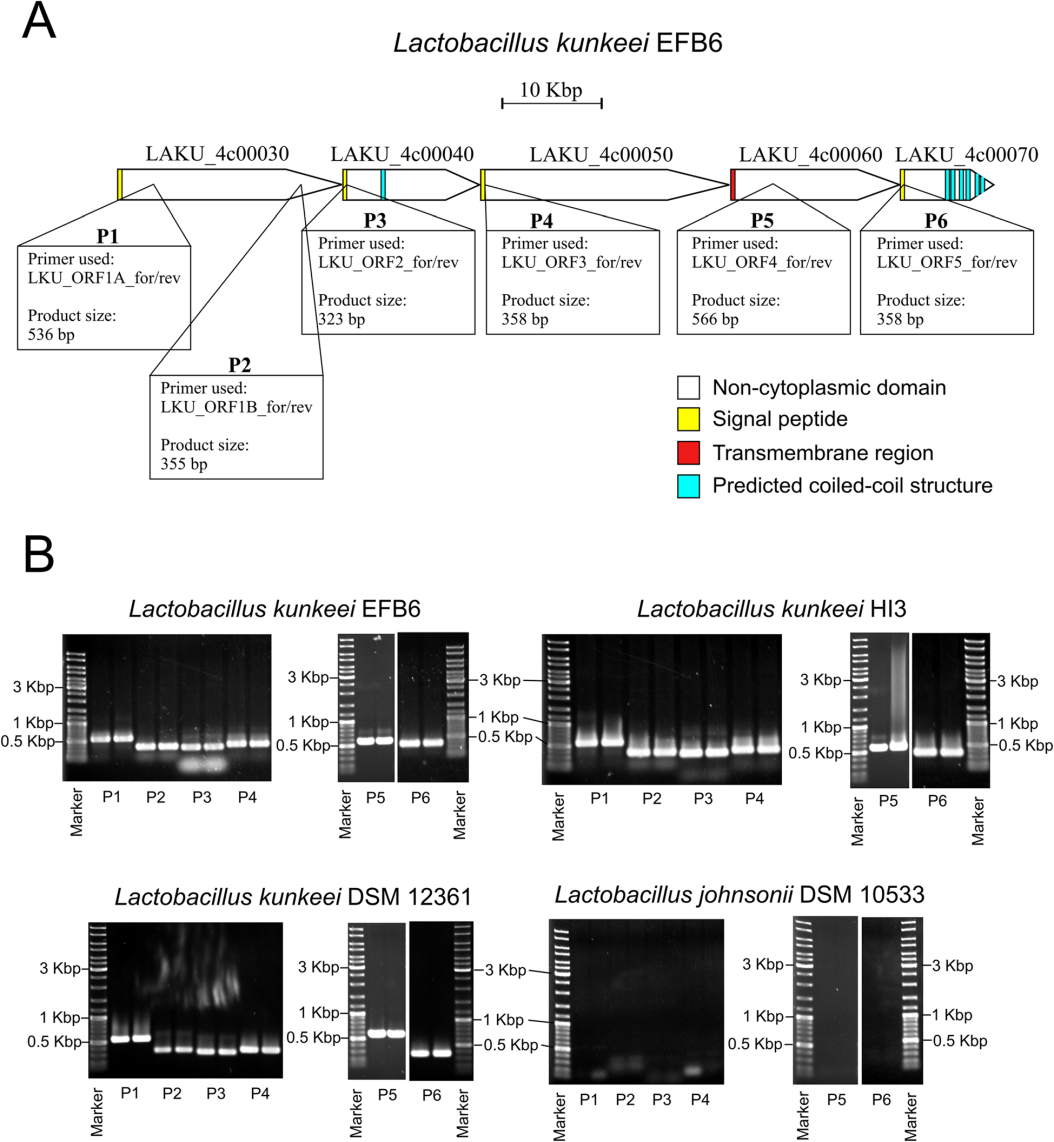
**Domain prediction (A) of the 89-kb region found in *L. kunkeei *EFB6 and its presence in other lactobacilli (B).** A combined transmembrane topology and signal peptide predictor [41] was used to determine putative domains. The yellow blocks represent signal peptides, the white color of the arrows show the non-cytoplasmic part. Red blocks represent transmembrane regions and blue blocks predicted coiled-coil structures. To test whether this region exists in other *L. kunkeei* strains, we designed specific primer-pairs for each ORF (Table 5, Figure 4A). Predicted PCR product sizes are depicted in white boxes. The presence of the genes were tested for *L. kunkeei* EFB6, *L. kunkeei* HI3 (isolated from honey), *L. kunkeei* DSM 12361 (isolated from wine), and *L. johnsonii* DSM 10533 (isolated from human blood) (Figure 4B). The obtained PCR product sizes correlated with the predicted sizes (Table 5, Figure 4A). For *L. johnsonii* DSM 10533, no PCR product could be obtained.

Analysis of the 89-kb region shown in Figure 3 revealed five ORFs (LAKU_4c00030-LAKU_4c00070) without orthologs in any genomes derived from lactobacilli deposited in GenBank (as of 28.02.2014). Furthermore, no homologs could be identified in any other sequenced microbial genome (NCBI nr-database as of 05.03.2014) by using blastp (e-value cutoff of 1e-20). Except for LAKU_4c00060 (7,521 amino acids), we could identify an N-terminal signal peptide and a non-cytoplasmic domain (Figure 4A) using Phobius’ domain prediction software [41]: LAKU_4c00040 (4,579 amino acids) and LAKU_4c00070 (3,129 amino acids) contain coiled coil structures. Except of LAKU_4c00050 (8,342 amino acids), all ORFs show weak similarity to large surface proteins or extracellular matrix-binding proteins found in bacteria such as *Staphylococcus*, *Streptococcus*, *Burkholderia*, *Weissella*, *Mannheimia*, and *Marinomonas*, but also in *Lactobacillus* and *Pediococcus*. Since, *L. kunkeei* EFB6 is the first sequenced genome harboring these cluster, we designed specific primer pairs for detection of each ORF in other *Lactobacillus* strains by PCR (Table 6). As shown in Figure 4B, all five ORFs were present in other *L. kunkeei* strains isolated from honey and wine. On the basis of domain prediction and IMG’s bidirectional best hits [32], we assume that this gene cluster encodes cell surface or secreted proteins involved in cell adhesion or biofilm formation.

**Figure 5:**
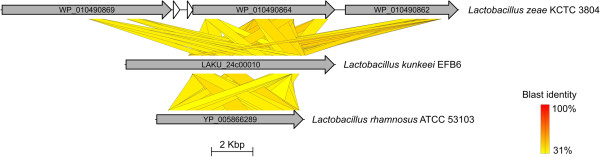
**Tblastx comparison of *L. kunkeei *ORF LAKU_24c00010 to matrix binding proteins of *L. rhamnosus *ATCC 53103 and “ *L. zeae *” KCTC 3804.** The graphical presentation was done with Easyfig software (minimum blast hit length of 200 bp and a maximum e-value of 1e^-100^) [44]. LAKU_24c00010 shows similarities to WP_010490869, WP_010490864 and WP_010490862 of “*L. zeae*” KCTC 3804, but also to YP_005866289 (*L. rhamnosus* ATCC 53103). The ORFs used for comparison are labeled with NCBI accession numbers. The blast identity is shown in a colored scale ranging from 31 % (yellow) to 100 % (red).

During genome comparison, we identified two additional proteins (LAKU_24c00010 and LAKU_24c00050) without a homolog in any of the publicly available genome sequences. These proteins show only weak sequence similarity to known proteins and might be involved in cellular adhesion. LAKU_24c00010 contains a signal peptide, transmembrane helices and 29 DUF1542 domains, which are typically found in cell surface proteins. In *Staphylococcus aureus*, it has been shown that some DUF1542-containing proteins are involved in cellular adhesion and antibiotic resistance [42]. LAKU_24c00010 showed the highest sequence identities to the matrix-binding protein (WP_010490864) of “*Lactobacillus zeae*” KCTC 3804 (40%) [43] and the extracellular matrix binding protein (YP_005866289) of *Lactobacillus rhamnosus* ATCC 53103 (36%) (Figure 5).

**Figure 6:**
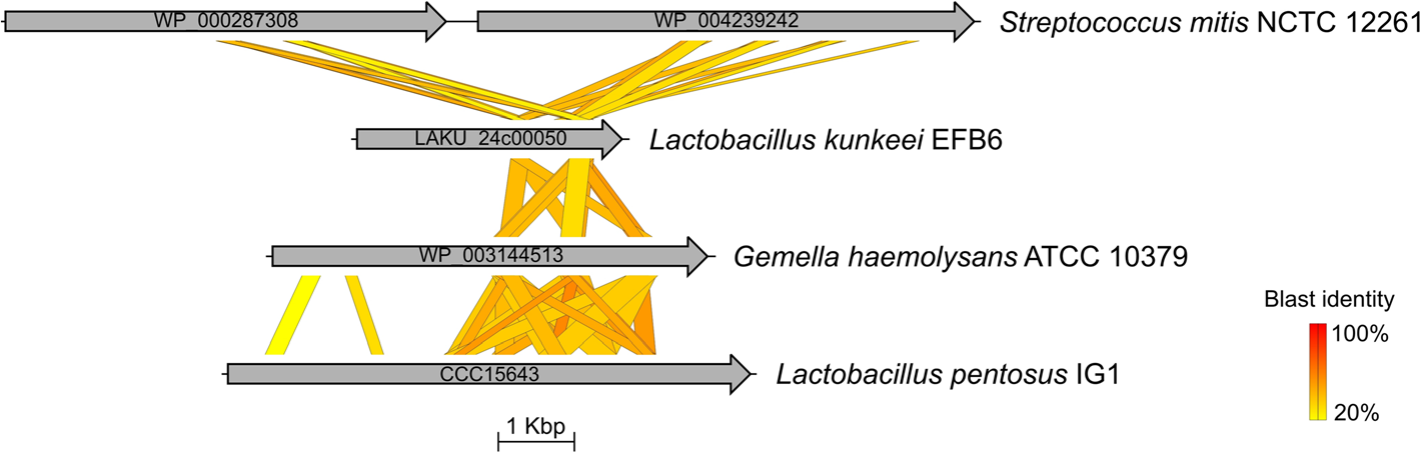
**Tblastx comparison of MucBP domain-containing proteins.** Comparison of MucBP domain-containing proteins were performed using the program Easyfig (mininum blast hit length of 50 bp and maximum e-value of 1e^-10^) [44]. LAKU_24c00050 shows similarity to ORFs of *Streptococcus mitis* NCTC 12261 (NCBI accession numbers inside arrows, which represent ORFs used for comparison). Additionally, LAKU_24c00050 shows similarity to WP_003144513 of *Gemella haemolysans* ATCC 10379 and CCC15643 of *Lactobacillus pentosus* IG1 [46]. The blast identity is shown in a colored scale ranging from 20% (yellow) to 100% (red).

Additionally, LAKU_24c00050 contains N terminal transmembrane helices, two mucin-binding protein domains as well as a C terminal Gram positive-anchoring domain. Proteins with this domain combination are usually associated with bacterial surface proteins. LAKU_24c00050 showed similarity to the Mlp protein (WP_004239242) of *Streptococcus mitis* and other mucus-binding proteins (Figure 6). Due to the mucosal surface-colonizing properties of lactobacilli, they have been investigated as potential recombinant mucosal vaccines [45].

In the genome of *L. kunkeei* EFB6, we identified genes encoding all proteins of the general secretory (Sec) pathway and putative polysaccharide biosynthesis proteins, which may participate in capsule or S layer formation. Recently, Butler et al. (2013) [47] detected a lysozyme produced by *L. kunkeei* Fhon2N and suggested a bacteriolysin or class III bacteriocin function. In *L. kunkeei* EFB6, we identified four genes belonging to the glycoside hydrolase family 25. Enzymes of this family are known to possess lysozyme activity. Two of the deduced proteins (LAKU_13c00160 and LAKU_32c00010) contain a signal peptide, indicating secretion of the proteins. LAKU_19c00290 harbors transmembrane helices and is probably anchored in the cell wall. LAKU_6c00080 did not contain a putative signal peptide or transmembrane helices.

### Rapid test PCR

Specific primer pairs have been designed to test other strains by PCR for the presence of an 89 kb region, which harbors five open reading frames (ORFs). Genomic DNA of the *L. kunkeei* strains EFB6, HI3 and DSM 12361, and *Lactobacillus johnsonii* DSM 10533 was used as template for PCR amplifications employing the thermal cycler peqSTAR 2X (PEQLAB Biotechnologie GmbH, Erlangen, Germany). PCR amplification was performed with the BIO-X-ACT™ Short DNA Polymerase (Bioline, Luckenwalde, Germany) and an initial denaturation step at 98°C for 2 min, followed by 30 cycles of denaturation for 20 s at 96°C, annealing for 20 s at 60°C and elongation for 30 s at 68°C. Subsequently, a final elongation step of 10 min at 68°C was performed. PCR products were purified employing the QIAquick PCR Purification Kit (Qiagen, Hilden, Germany).

## Conclusion

In this study, we characterized the genome of *L. kunkeei* strain EFB6 isolated from an EFB-diseased larva. In a recent study was shown that *L. kunkeei* has the potential for biofilm formation and adhesion to the honey crop [6]. Our genome analysis supports these results. Using large surface proteins or extracellular matrix-binding proteins, *L. kunkeei* might be able to attach to eukaryotic epithelial cells. Furthermore, due to the presence of polysaccharide biosynthesis proteins and several enzymes with lysozyme activity, it is possible that *L. kunkeei* is actively protecting its niche against bacterial competitors. As LABs have been shown to have an inhibitory growth effect on *M. plutonius*, the use of LABs as probiotic additive against the EFB-causing agent is conceivable.

## Abbreviations

AFB: American foulbrood of honeybees; EFB: European foulbrood of honeybees; LABs: Lactic acid bacteria; TEM: Transmission electron microscopy.

## Competing interests

The authors declare that they have no competing interests.

## Authors’ contributions

MD, AP and RD designed research, MD, JS and FJT isolated and characterized strain EFB6, MD, AP and AL carried out genome analyses, MH performed electron microscopy, MD and RD wrote the manuscript with help of AP. All authors read and approved the final manuscript.

## Supplementary Material

Additional file 1 Associated MIGS Record. Click here for file
