# Supplementary material for: High quality draft genome of Lactobacillus kunkeei EFB6, isolated from a German European foulbrood outbreak of honeybees
Source: Stand Genomic Sci. 2015 Feb 19;10:16. doi: 10.1186/1944-3277-10-16 (PMC4511666; doi:10.1186/1944-3277-10-16)
Supplement: Additional file 1 — Associated MIGS Record. [file 1944-3277-10-16-S1.docx]

# Associated MIGS Record

# Table S1. Associated MIGS record

| **MIGS-ID** | field name | description |
| --- | --- | --- |
| **MIGS-1** | Submit to INSDC/Trace archives | AZBY00000000 |
| **1.1** | PID | 227106 |
| **1.2** | Trace Archive | SRR1502204 |
| **MIGS-2** | MIGS CHECK LIST TYPE |  |
| **MIGS-3** | Project Name | [*Lactobacillus kunkeei*](http://dx.doi.org/10.1601/nm.5393) EFB6 |
| **MIGS-4** | Geographic Location | Bavaria, Germany |
| **4.1** | Latitude | 49°14' N |
| **4.2** | Longitude | 11°05' E |
| **4.3** | Depth |  |
| **4.4** | Altitude | 400 m a.s.l |
| **MIGS-5** | Time of Sample collection | 2012-10-1 |
| **MIGS-6** | Habitat (EnvO) | terrestrial |
| **6.1** | temperature |  |
| **6.2** | pH |  |
| **6.3** | salinity |  |
| **6.4** | chlorophyll |  |
| **6.5** | conductivity |  |
|  |  |  |
| **6.6** | light intensity |  |
| **6.7** | dissolved organic carbon (DOC) |  |
| **6.8** | current |  |
| **6.9** | atmospheric data |  |
| **6.10** | density |  |
| **6.11** | alkalinity |  |
| **6.12** | dissolved oxygen |  |
| **6.13** | particulate organic carbon (POC) |  |
| **6.14** | phosphate |  |
| **6.15** | nitrate |  |
| **6.16** | sulfates |  |
| **6.17** | sulfides |  |
| **6.18** | primary production |  |
| **MIGS-7** | Subspecific genetic lineage |  |
| **MIGS-9** | Number of replicons |  |
| **MIGS-10** | Extrachromosomal elements |  |
| **MIGS-11** | Estimated Size | 1566851 |
| **MIGS-12** | Reference for biomaterial or Genome report | [10] |
| **MIGS-13** | Source material identifiers |  |
| **MIGS-14** | Known Pathogenicity |  |
|  |  |  |
| **MIGS-15** | Biotic Relationship | Host-associated |
| **MIGS-16** | Specific Host | Apis mellifera |
| **MIGS-17** | Host specificity or range (taxid) | 7460 |
| **MIGS-18** | Health status of Host | Honeybee larva infected with European foulbrood |
| **MIGS-19** | Trophic Level | Heterotroph |
| **MIGS-22** | Relationship to Oxygen | Facultative |
| **MIGS-23** | Isolation and Growth conditions | MRS medium (Carl Roth GmbH & Co KG, Karlsruhe, Germany), 35°C, aerobic conditions |
| **MIGS-27** | Nucleic acid preparation | Epicentre® MasterPureTM DNA Purification kit (Epicentre®, Madison, WI, USA) |
| **MIGS-28** | Library construction |  |
| **28.1** | Library size | 1 kb |
| **28.2** | Number of reads | 2000000 |
| **28.3** | vector |  |
| **MIGS-29** | Sequencing method | Illumina |
| **MIGS-30** | Assembly |  |
| **30.1** | Assembly method | SPAdes 2.5 |
| **30.2** | estimated error rate |  |
| **30.3** | method of calculation |  |
| **MIGS-31** | Finishing strategy |  |
| **31.1** | Status | Permanent draft |
| **31.2** | coverage | 143x |
| **31.3** | contigs | 55 |
| **MIGS-32** | Relevant SOPs |  |
| **MIGS-33** | Relevant e-resources | GenBank (http://www.ncbi.nlm.nih.gov/nuccore/AZBY00000000), SeqCentre: Goettingen Genomics Laboratory, DOE Joint Genome Institute (https://img.jgi.doe.gov/cgi-bin/er/main.cgi?section=TaxonDetail&page=taxonDetail&taxon_oid=2558860143) |
